# Quantitative Assessment of CFD-Based Micro-Scale Renovation of Existing Building Component Envelopes

**DOI:** 10.3390/biomimetics10110733

**Published:** 2025-11-01

**Authors:** Yan Pan, Lin Zhong, Jin Xu

**Affiliations:** 1 School of Design and Art, Jiangxi University of Finance and Economics, Nanchang 330031, China; panyan0701@126.com; 2 School of Jewelry, West Yunnan University of Applied Sciences, Dali 671000, China; zhonglinhanyang@gmail.com

**Keywords:** existing buildings, facade component envelope structure, bionic design, CFD simulation, urban micro-renewal, quantitative evaluation

## Abstract

With the acceleration of urbanization, environmental degradation is increasingly restricting the improvement of residents’ quality of life, and promoting the transformation of old communities has become a key path for sustainable urban development. However, existing buildings generally face challenges, such as the deterioration of the performance of the envelope structure and the rising energy consumption of the air conditioning system, which pose a serious test for the realization of green renovation. Inspired by the application of bionics in the field of architecture, this study innovatively designed five types of bionic envelope structures for outdoor air conditioning units, namely scales, honeycombs, spider webs, leaves, and bird nests, based on the aerodynamic characteristics of biological prototypes. The ventilation performance of these structures was evaluated at three scales—namely, single building, townhouse, and community—under natural ventilation conditions, using a CFD simulation system. The study shows the following: (1) the spider web structure has the best comprehensive performance among all types of enclosures, which can significantly improve the uniformity of the flow field and effectively eliminate the low-speed stagnation area on the windward side; (2) the structure reorganizes the flow structure of the near-wall area through the cutting and diversion of the porous grid, reduces the wake range, and weakens the negative pressure intensity, making the pressure distribution around the building more balanced; (3) in the height range of 1.5–27 m, the spider web structure performs particularly well at the townhouse and community scales, with an average wind speed increase of 1.1–1.4%; and (4) the design takes into account both the safety of the enclosure and the comfort of the pedestrian area, achieving a synergistic optimization of function and performance. This study provides new ideas for the micro-renewal of buildings, based on bionic principles, and has theoretical and practical value for improving the wind environment quality of old communities and promoting low-carbon urban development.

## 1. Introduction

With the continuous growth of global urbanization, a large number of rural populations have migrated to cities, and the demand for urban housing has increased significantly [1]. China’s urbanization rate has risen from 17.9% in 1978 to 67% at the end of 2024, and it has now fully entered the middle and late stage of transformation. However, most of the existing residential buildings were built around 2000, or even earlier, and were subject to the poor economic foundation and building standards at that time. There are problems such as structural aging, increased energy consumption, and damage to the built environment [2]. In particular, there are still a large number of residential buildings built in the 1950s–1980s that have been completed and used for nearly or more than 50 years, and there are serious safety hazards [3]. Therefore, the focus of urban development at this stage will shift from large-scale demolition and expansion to the maintenance and renovation of existing spaces [4], and the renewal of old communities has become the best carrier for achieving sustainable development goals. 

The environmental problems exposed by the rapid development of cities are increasingly affecting the quality of life of residents, such as poor environmental ventilation performance, poor air quality, and a large amount of energy consumption, which are important factors affecting sustainability [5,6]. Globally, buildings account for about 40% of energy consumption and 33% of greenhouse gas emissions [7,8]. In this context, the EU has mainly relied on the Energy Efficiency Directive (EED) 2012/27/EU and the Energy Performance of Buildings Directive (EPBD) 2010/31/EU to improve the energy performance of buildings in the past 15 years, and revised the EPBD in 2023 to achieve energy-saving goals [9,10]. According to the report “Carbon Emissions in China’s Urban and Rural Construction (2024 Edition)”, released by the China Association for Building Energy Efficiency (CABEE), the life-cycle energy consumption of Chinese buildings accounts for 44.8% of the total national energy consumption and 48.3% of carbon emissions. Therefore, the Chinese government has identified building energy conservation as a key area in the 14th Five-Year Plan, from energy-saving buildings to green buildings, and from single buildings to regional buildings [11]. Related research has grown by 650% since 2018, showing a significant increase [12]. However, most scholars tend to focus on new buildings, and how to sustainably renovate existing buildings and existing built environments is an urgent problem to be solved. 

Existing studies have shown that factors such as aging of the envelope structure of existing buildings, limited vegetation, and obstructed natural ventilation can reduce the thermal comfort of residents in dense building environments, and excessive reliance on mechanical ventilation can also lead to a surge in energy consumption [13,14,15]. Among various mitigation strategies, urban ventilation is considered to be a natural and energy-saving solution, which can reduce energy demand by 6.704% by improving ventilation [16,17,18]. Lee, Li, N, Lyu, Y et al. believe that building orientation, tree layout, and street width play a key role in shaping the wind environment of existing spaces [19,20,21]. However, due to the large number of old buildings that need to be renovated, the scale and investment consumed by macro-renovation projects such as adjusting building orientation and layout are huge, and they do not meet the sustainable development goals in terms of economy and environment.

**Figure 1:**
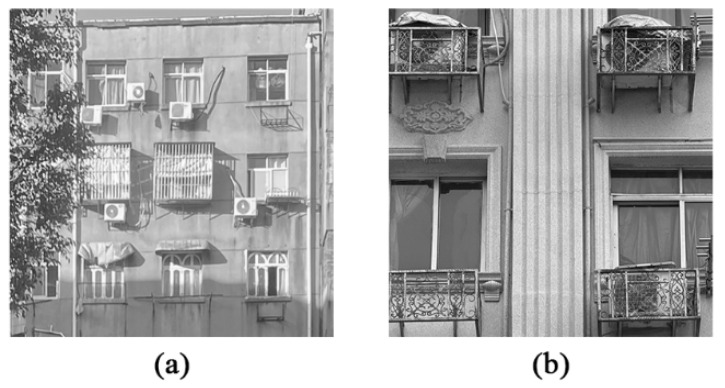
Current situation of air conditioning external units in old communities. (**a**) No enclosure structure and (**b**) enclosed structure.

Therefore, it is more feasible to optimize the ventilation of old communities at the micro level. Old houses have problems such as closed balconies, anti-theft cages on the outer windows, and cluttered external decorative components such as air-conditioning brackets [3], as shown in Figure 1. Quan et al. [22] analyzed the wind-pressure distribution of external decorative structures such as horizontal cantilevered sunshades and vertical ribs, and their impact on the wind load of building facades. Yuan et al. [23] analyzed the effects of 21 facade exterior decorative structures on the extreme wind pressure of the building facade. Shen et al. [24] believed that the external hollow decorative structure had a significant weakening effect on the average and pulsating wind pressure of the crosswind direction. Combined with the content requirements of the comprehensive renovation project of the old residential district [3], as shown in Table 1, it is found that the design of the existing building facade structure of the air conditioning outdoor unit enclosure can promote the sustainability of the old residential district renovation in a low-cost and high-efficiency manner, but previous studies lack verification in understanding how the air conditioning outdoor unit enclosure structure affects the wind environment of the old residential district.

In the field of urban and architectural design, the concept of bionics has been widely applied to solve global environmental problems [25,26]. Bionic methods can address these challenges on multiple scales, from individual mechanical units (materials), to buildings, to entire urban areas [27,28,29]. The term bionics was proposed by biologist Janine Benyus in 1997, defining “interdisciplinary collaboration between biology and technology or other innovative fields, through functional analysis of biological systems, abstracting them into models, and transferring these models to solutions and applying them” [30]. Bionics methods are usually divided into two categories: defining human design needs or problems and finding solutions from other organisms or ecosystems to solve these problem, or identifying specific characteristics, behaviors, or functions in organisms or ecosystems and transforming them into human designs. This use of nature and organisms can be achieved in three ways: replication, abstraction, and inspiration [31,32]. There are three levels of imitation in architecture, including the imitation of biological characteristics, behaviors, and ecosystems [33]. Compared with other methods, the bionics method has unique advantages in ecological design and sustainability, and can combine nature with architecture and environmental design to improve energy efficiency and transform the urban environment into a healthier and more climate-adaptive space, to meet the needs of citizens [34,35,36,37,38]. Therefore, this study will guide the design of the enclosure structure of the old community, based on the theory of bionics.

In the process of bionic architecture research, the use of biological structures for wind environment optimization is as follows [25]. Both the Malaysian architectural bionics research and the architectural entity of the East Gate Center in Harare, Zimbabwe, emphasized that the application of the passive ventilation system of the termite mound morphology to high-rise buildings can significantly reduce the energy consumption and carbon footprint of buildings [39,40,41]; Pugalenthi et al. [42] analyzed how the honeycomb structure and natural ventilation system optimized the thermal efficiency and energy efficiency of buildings. Gao et al. [43] also drew inspiration from the honeycomb structure of plant cells to design a bionic vertical greening bracket on the facade of Suzhou buildings, which effectively reduces the urban heat island effect while maintaining cultural continuity. Han et al. [44,45] found that the building surface structure, designed by imitating plant leaves, can reduce urban heat islands and weaken noise. Castriotto C et al. [46] believe that a bird’s nest design is a source of inspiration for the strength and lightness of buildings, and the winding arrangement of branches optimizes natural ventilation and lighting; Regassa Y [47] applies the spider web structure to the design of building facades and flexible roofs, which is used to optimize passive ventilation and improve the structural performance of buildings. Othmani et al. [32,48] used the scale structure as a reference for the design of building facades to optimize passive ventilation and solar reflection in order to improve indoor and outdoor thermal comfort; the London “Gherkin” used the bionic design of the Venus’ flower basket sponge (glass sponge) to reduce wind resistance and improve the airflow around the building by using aerodynamic shape, while optimizing the structural performance of the building [49], as shown in Table 2.

Among the seven biological structures summarized above, the termite mound is a typical case of improving the indoor wind environment by relying on the internal connectivity of the building. Its benefits depend on the interaction of the overall structure of the building. This study cannot extract its characteristics and directly apply them to the envelope structure. The biological characteristics of the Venus’ flower basket sponge are difficult to standardize. If it is only abstracted as a simple cylinder, it is difficult to reflect its bionic significance. The case is also applied to the whole building. The five categories of honeycomb, leaf, bird’s nest, spider web, and fish scales are all biological characteristics that can be applied to the facade, frame, and other covering structures of the building. (1) The blade has the function of guiding and reducing resistance: it smoothly guides the wind flow through its streamlined shape, effectively avoiding airflow separation, thereby significantly reducing the wind load and downstream turbulence; (2) the honeycomb has the function of diversion and stability: it uses a dense unit structure to divide the atmospheric flow into countless stable small eddies, greatly dissipating wind energy and homogenizing wind pressure, and improving structural stability; (3) the bird’s nest has the function of penetration and buffering: as a porous medium, it allows part of the wind flow to penetrate and consume its energy through internal friction, and at the same time, the remaining airflow flows around, thereby reducing wind resistance and wind speed by “sparse” instead of “blocking”; (4) the fish scales have a dynamic response effect: they have the ability to passively open and close according to the wind conditions, thereby dynamically adjusting the roughness and ventilation rate of the building surface, and achieving adaptive unloading and ventilation at different wind speeds; (5) the spider web has the function of filtering and energy dissipation: it uses its ultra-fine fibers to vibrate in the wind to dissipate a large amount of wind energy, which can effectively divide a large-scale vortex, and play an efficient filtering and vibration reduction effect. Therefore, the selection of these five types of biological forms can meet the needs of this study for the bionic design of the external machine enclosure structure. 

With the improvement of computer simulation and data processing capabilities, the use of computational fluid dynamics (CFD) technology to integrate the natural ventilation design into the architectural design and optimization stage has become mainstream in natural ventilation research. This method can be more intuitive and lower in cost. A visualization of the CFD numerical simulation results is seen in [50]. Loche I et al. [51] used CFD to simulate the effects of building balcony depth, openings, railing grilles, etc., on local wind speed, pressure distribution, and facade ventilation; Suárez M [52] used CFD to study how the arrangement of building facade decorations/panels affects the natural ventilation speed and energy consumption of the building cavity, and gave suggestions for optimizing the arrangement. Tai V C [53] et al. used CFD to study the effect of external louvers on the crosswind ventilation performance of buildings at different blade angles and positions; Alyahya A [54] et al. used bionic principles to design a ventilation curtain wall system, and used CFD to verify the performance of 13 bionic interior configurations in natural ventilation and heat dissipation. Therefore, this study uses CFD simulation to simulate the wind environment of old buildings and analyzes the ventilation performance of the bionic envelope based on data indicators.

In summary, this study will focus on the optimization of the enclosure structure of the outdoor air conditioner in the old community, extract the biological structure characteristics that can improve the wind environment, design the prototype, and conduct the CFD simulation tests. The study will set up the following CFD simulation tests for the ventilation benefits of the envelope structure from the micro- to the mesoscale: first, the original air conditioning outdoor unit bracket, without the envelope structure, and the five types of bionic structures will be compared on the single scale to verify which type of bionic structure is optimal in a simple wind environment. A townhouse house model will be designed with this method to verify the structural ventilation under the corridor effect. According to the 12 sets of experimental data, the spider web structure is the most optimal. Finally, the CFD simulation will be performed on the community scale, with the original structure and without the envelope structure, to explore the bionic design method that can optimize the complex wind environment. It is worth noting that this study mainly utilizes the advantages of PHOENICS (2019 version) computational fluid dynamics (CFD) simulation software developed by CHAM, a company in London, UK (acquired by Zhongwang Software in 2023), focusing on the wind speed and wind pressure differences in the bionic structure to optimize the wind environment, while the unit heat dissipation, energy consumption, and other issues need to be further studied in subsequent research.

## 2. Materials and Methods

### 2.1. Research Object

#### 2.1.1. Existing Building and Outdoor Air Conditioning Unit Model

The CMAB dataset [55] covers all 3667 spatial cities in mainland China, covering more than 31 million individual buildings. By filtering the “age” attribute in the CMAB dataset, see Table 3, buildings with a building date within the range of 1995–2005 are extracted, and the existing building dimensions are determined in combination with the “Residential Design Code” (GB 50096-2011, China) [56] and “Real Estate Measurement Code” (GB/T 17986.1-2000) [57], issued around 2000. The CAARC (Commonwealth Advisory Aeronautical Research Council) is a standard building model that is widely used in the field of wind engineering. This model is often used to test the accuracy of wind tunnel test techniques and methods, and is also used by many scholars as a benchmark model for building wind environment research [58]. Based on the actual building size data, and in order to simplify the simulation difficulty, the simulated experimental building is set to a 12 m × 10 m × 27 m, mid-rise rectangular model with a floor height of 3 m [39].

**Figure 2:**
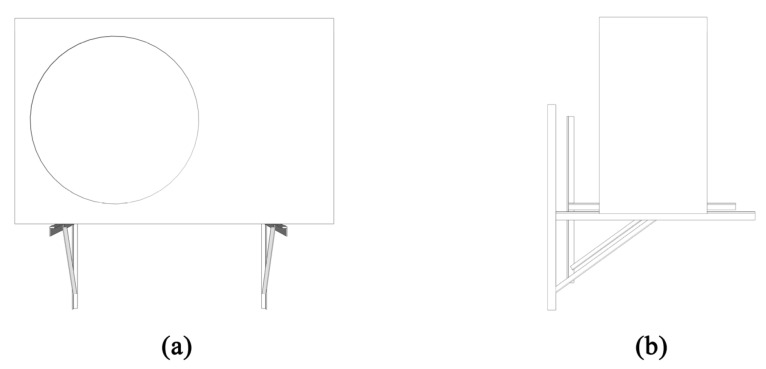
Standardized modeling of the original 1.5 horsepower air conditioning outdoor unit. (**a**) Front view; (**b**) Side view.

The original air conditioner bracket in the old community only plays a supporting role and has no diversion effect. The size of the 1.5 horsepower outdoor unit produced by each air conditioner manufacturer was studied and sorted out, and the outdoor unit of the air conditioner was uniformly set to a rectangular model of 0.85 m × 0.55 m × 0.3 m, and the frame size of the enclosure structure was set to 1.3 m × 0.6 m × 0.6 m, as shown in Figure 2.

#### 2.1.2. Bionic Envelope Model of the Air Conditioner Outdoor Unit

**Figure 3:**
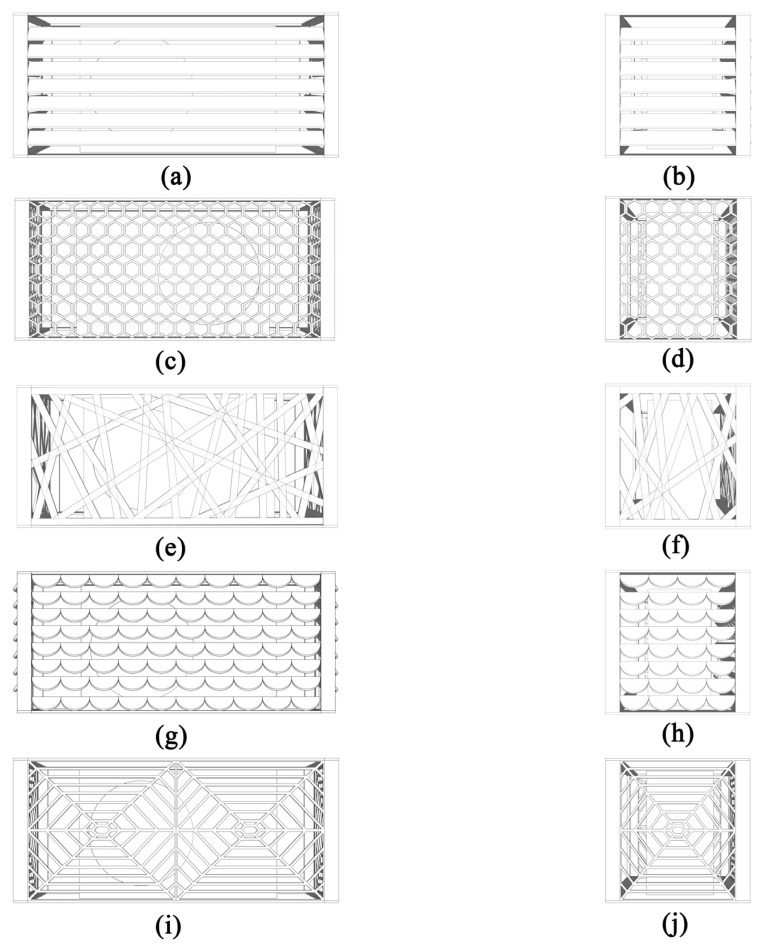
Bionic design of the outer machine enclosure structure: (**a**) front of the blade structure; (**b**) side of the blade structure; (**c**) front of the honeycomb structure; (**d**) side of the honeycomb structure; (**e**) front of the bird’s nest structure; (**f**) side of the bird’s nest structure; (**g**) front of the fish scale structure; (**h**) side of the fish scale structure; (**i**) front of the spider web structure; and (**j**) side of the spider web structure.

After the literature and case screening, the biological structures that can optimize the ventilation efficiency of the external support of the building facade mainly include leaf-like, honeycomb-like, bird’s nest-like, scale-like, and spider web-like structures. In the design process, it is necessary to ensure that the effective ventilation area of the air conditioning outdoor unit enclosure is not less than 60%. The original non-enclosure structure bracket is made of 4 pieces of 600 mm-long and 2 pieces of 400 mm-long 24 mm × 24 mm high angle steel welding, as shown in Figure 2; the leaf-like bionic structure is a common enclosure decoration in the renovation of old communities and new communities. The study includes it in the simulation experiment to compare and explore whether the ventilation efficiency is better than the leaf-like bionic structure. The front blade size is 1200 mm × 65 mm, and the side blade is 480 mm × 65 mm, as shown in Figure 3a,b; the honeycomb bionic structure is inspired by the beehive, and it is composed of a hexagonal structure with a side length of 40 mm, and both sides are connected to the frame, as shown in Figure 3c,d; the bird’s nest-like bionic structure is more special, and the study extracts the composition form of the Beijing Bird’s Nest Gymnasium, and the curved branches are flattened and crossed, which is a non-standard structure, as shown in Figure 3e,f; the scale-like bionic structure is extracted from the skin surface of fish, snakes, etc., and it is composed of a semicircular arc with a diameter of 123 mm, as shown in Figure 3j,h; the front of the spider web-like bionic structure is spliced by two 580 mm × 615 mm mesh rectangles and the side is embedded by a 580 mm × 480 mm mesh rectangle, and the spider webs on both sides are spread from the center point to the surroundings, and the internal line width is 10 mm, as shown in Figure 3i,j; all the above bionic shapes have a thickness of 5 mm.

**Figure 4:**
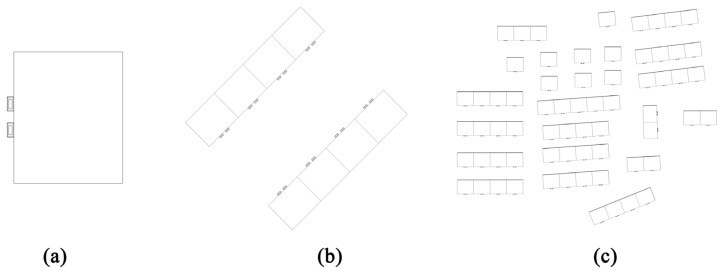
Three-scale old building wind environment modeling: (**a**) single building; (**b**) townhouse; and (**c**) old community.

In order to verify the efficiency of the design of the bionic envelope structure of the air conditioner outdoor unit on the improvement of the wind environment, after obtaining the standard building model and the bionic structure model, three types of volumes—namely, single building, townhouse building, and typical community—were used for simulation and comparison experiments. The single building is a rectangular simplified model with a size of 12 m × 10 m × 27 m. The outdoor unit of the air conditioner is placed on the north side of the building, and the height from the ground is 1.9 m, as shown in Figure 4a. At this scale, the independent ventilation efficiency of each bionic structure can be verified; the single side of the townhouse building is spliced from four single buildings, and the single column size is 48 m × 10 m × 27 m. The outer machine enclosure structure is set on the side walls on both sides of the corridor, the building spacing is 25 m, and the building orientation is 135°, as shown in Figure 4b. At this scale, the ventilation efficiency of the simple street form of the bionic structure can be verified; the typical community selects the typical old community characteristics of Donghu District, Nanchang City, Jiangxi Province for the simulation. The community covers an area of about 28,500 m^2^, and consists of a group of five units, 11 groups of four units, and a group of three units. The townhouse building, a combined building consisting of 3 groups of two buildings, and eight single buildings, as shown in Figure 4c, verify the comprehensive ventilation efficiency of the bionic structure. 

### 2.2. Research Process

**Figure 5:**
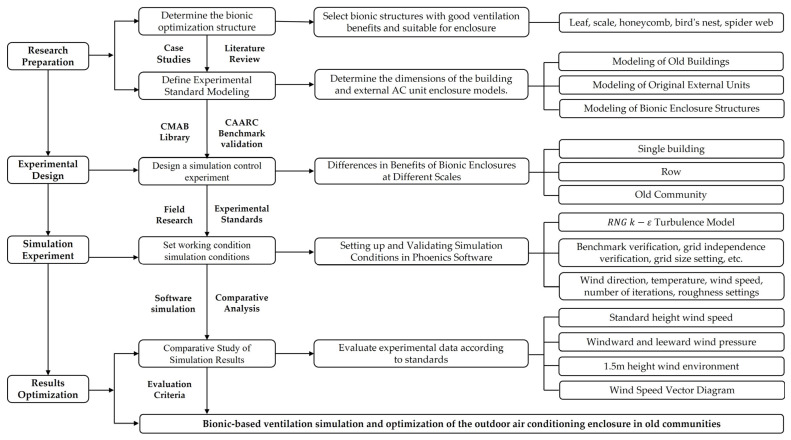
Research framework.

First, the natural organisms with optimized architectural wind environment attributes are sorted out, and the structural forms that are suitable for bionics are selected in combination with the research objects. Then, the five bionic structures and old buildings are standardized according to the policy documents and design specifications. At the same time, the influence of the building’s body shape on the wind environment is simplified to further explore the performance differences in the five types of bionic structures. Secondly, after completing the grid independence verification and the calculation of the domain setting, and verifying the simulation accuracy through the benchmark case, all working conditions are set and calculated in detail to ensure the reliability of the research results. Then, CFD simulation was performed on the three scales of the standardized building model, and the differences between the five types of structures in different building environments were explored through the comparison of experimental data. Finally, the evaluation criteria set by the study were combined to determine the optimal model structure type for the natural ventilation efficiency of the outdoor unit enclosure structure of the old building’s air conditioner. The research framework is shown in Figure 5.

### 2.3. Research Methods and CFD Simulation Details

#### 2.3.1. Evaluation Criteria

The evaluation criteria for the wind environment of the community in this study refer to the international authoritative standards “Green Building Evaluation System” (GB/T 50378-2019, China) [59] and “Code for Design of Heating, Ventilation and Air Conditioning of Civil Buildings” (GB 50736-2012, China) [60], as well as the standards established by some scholars [61], mainly including the following contents:

- The street environment at a pedestrian height of 1.5 m is set as the lowest measurement section, and the average wind speed of different floors is analyzed as an evaluation index. Among them, the wind speed of 1–3 m/s, not exceeding 5 m/s, is usually more appropriate.
- According to the specifications and previous research, the wind pressure difference exceeding 3 pa between the windward and leeward sides of the building is used as the evaluation standard for natural ventilation.
- This study uses CFD simulation data of standard building models of 3 m, 9 m, 15 m, 21 m, and 27 m standard floors, combined with a site wind speed map, wind pressure map, and wind speed distribution vector map of 1.5 m pedestrian height as the main evaluation basis.

#### 2.3.2. CFD Simulation Set Up

CFD simulation software mainly includes (1) general software: Phoenics (2019 R1), Fluent (2019 R1), scSTREAM (2019); (2) built-in tools in the design platform: Autodesk CFD (2020); (3) parametric modeling platform: Rhino 7 (with CFD plug-ins such as Grasshopper and Butterfly). Among them, PHOENICS, as the world’s first commercial computational fluid dynamics (CFD) software, has a built-in building module (FLAIR) that supports indoor and outdoor wind environments, thermal comfort (PMV/PPD), pollutant diffusion, solar radiation, and other special analysis, and is widely used in the field of architecture and urban planning [62]. Shang Y [63] used PHOENICS software to simulate the outdoor winter wind environment of the old community in Beijing, providing an objective basis for the improvement of the outdoor environment of the street; Yang Y [64] used PHOENICS software to simulate the wind environment of the building group in the Shanghai business district, providing a reference for the formulation of relevant planning and design standards. Zhang L [65] used PHOENICS software to establish a three-dimensional building model to simulate the thermal environment under different greening conditions, proving that green roofs can reduce building energy consumption. Jin Y [66] used a questionnaire survey and PHOENICS software simulation to study the summer ventilation environment of a university teaching building in a cold region; Liu X [67] used PHOENICS software to simulate and analyze the east coast of Jiaozhou Bay in Qingdao, and analyzed the influence mechanism of different morphological parameters on the summer wind environment under equal density conditions; Chen L [68] used PHOENICS software to perform a numerical simulation of wind environment parameters, such as summer wind speed and wind pressure, in the old residential area of Xicheng Street in Ya’an, and optimized the wind environment from four aspects: building layout, street space, public space, and greening configuration. In summary, PHOENICS software is suitable for the simulation study of the wind environment of community streets, and it can effectively verify the impact of changes in environmental factors on the wind environment parameters, and the calculation results are reliable. Therefore, this study uses PHOENICS software to explore the CFD simulation of the wind environment of old communities with bionic structures. The simulation settings are as follows.

Turbulent flow model equation: This study selected the Flair module of PHOENICS for simulation. There are two improved variants of the typical turbulent flow model for the external flow around the building, which are more suitable for the ventilation test of the external machine enclosure.

1. The standard k−ε turbulence model is a simple industrial flow field and heat transfer simulation, without a large pressure gradient, separation, and strong curvature flow, which is suitable for initial parameter research, and generally applicable to building ventilation. Its formula is shown in Equations (1) and (2):
  (1) $$\frac{\partial \left(\rho k\right)}{\partial t}+\frac{\partial \left(\rho ku_{i}\right)}{\partial x_{i}}=\frac{\partial }{x_{j}}\left(\alpha_{k}\eta_{eff}\frac{\partial k}{\partial x_{j}}\right)+G_{k}+\rho \varepsilon$$
  (2) $$\frac{\partial \left(\rho \varepsilon \right)}{\partial t}+\frac{\partial \left(\rho \varepsilon u_{i}\right)}{\partial x_{i}}=\frac{\partial }{\partial x_{j}}\left(\alpha_{\varepsilon }\eta_{eff}\frac{\partial \varepsilon }{\partial x_{j}}\right)+\frac{C_{1s}^{*}\varepsilon }{k}G_{k}-C_{2s}\rho \frac{\varepsilon^{2}}{k}$$
  where k is the turbulent kinetic energy; ε is the turbulent dissipation rate; ρ is the fluid density; and $G_{k}$ is the average velocity gradient generation term.

2. The Chen−Kim k−ε turbulence model is an improved variant of the standard k−ε model. An additional generation term $C_{3\varepsilon }\frac{\varepsilon }{k}G_{k}$ is added to the transport equation of the dissipation rate, ε. This additional generation term is designed to accelerate the generation of the dissipation rate, ε*,* when the flow energy generation, $G_{k}$, suddenly increases, so that the model can respond to flow changes more quickly. The formulas are shown in Equations (3) and (4):
  (3) $$\frac{\partial \left(\rho k\right)}{\partial t}+\frac{\partial \left(\rho ku_{j}\right)}{\partial x_{j}}=\frac{\partial }{\partial x_{j}}\left[\left(\mu +\frac{\mu_{i}}{\sigma_{k}}\right)\frac{\partial k}{\partial x_{j}}\right]+P_{k}-\rho \varepsilon$$
  (4) $$\frac{\partial \left(\rho \varepsilon \right)}{\partial t}+\frac{\partial \left(\rho \varepsilon u_{i}\right)}{\partial x_{i}}=\frac{\partial }{\partial x_{j}}\left(\alpha_{\varepsilon }\eta_{eff}\frac{\partial \varepsilon }{\partial x_{j}}\right)+\frac{C_{1s}^{*}\varepsilon }{k}G_{k}-C_{2s}\rho \frac{\varepsilon^{2}}{k}$$
  where $\mu_{t}$ is the turbulent viscosity coefficient; $P_{k}$ is the turbulent kinetic energy generated by the mean velocity gradient; and μ is the molecular viscosity coefficient.

3. The RNG k−ε turbulence model applies the renormalization group (RNG) theory to mathematically derive the revised equation. It includes the strain rate’s additional term and optimizes the model constants, which is more accurate in predicting the separated flow, moderate swirl, and shear flow, and the simulation accuracy of the complex flow is significantly higher than that of the standard k−ε model and Chen−Kim k−ε model, and its formula is shown in Equations (5) and (6):
  (5) $$\frac{\partial }{\partial t}\left(\rho k\right)+\frac{\partial }{\partial x_{i}}\left(\rho ku_{i}\right)=\frac{\partial }{{\partial x}_{i}}\left(\alpha_{k}\mu_{eff}\frac{\partial k}{\partial x_{j}}\right)+G_{k}+G_{b}-\rho \varepsilon -Y_{M}+S_{k}$$
  (6) $$\frac{\partial }{\partial t}\left(\rho \varepsilon \right)+\frac{\partial }{\partial x_{i}}\left(\rho \varepsilon u_{i}\right)=\frac{\partial }{\partial x_{j}}\left(\alpha_{\varepsilon }\mu_{eff}\frac{\partial \varepsilon }{\partial x_{j}}\right)+C_{1\varepsilon }\frac{\varepsilon }{k}\left(G_{k}+C_{3\varepsilon }G_{b}\right)-C_{2\varepsilon }\rho \frac{\varepsilon^{2}}{k}-R_{\varepsilon }+S_{\varepsilon }$$
  where GK represents the turbulent kinetic energy generated by the average velocity gradient; Gb represents the turbulent kinetic energy generated by buoyancy; $Y_{M}$ is the model correction term; $S_{k}$ is the turbulent kinetic energy source term; and R is the strain rate correction term.

Combining the characteristics of the simulation object and the amount of data in this study, the calculation process mainly focuses on the possible flow separation, local vortex, and complex airflow organization in the narrow space around the external machine components. This type of flow has a strong curvature effect and obvious shear characteristics, so it is necessary to select a model that can more accurately capture the anisotropic turbulent structure. The focus is on analyzing the optimization effect of the bionic envelope on the ventilation and heat dissipation efficiency of the outdoor air conditioner, and also simulating its external flow field structure and heat exchange characteristics without involving the details of indoor air convection. In order to improve the simulation accuracy and reasonably characterize the turbulent transport process, the RNG k−ε turbulence model was finally selected for numerical calculation.

**Figure 6:**
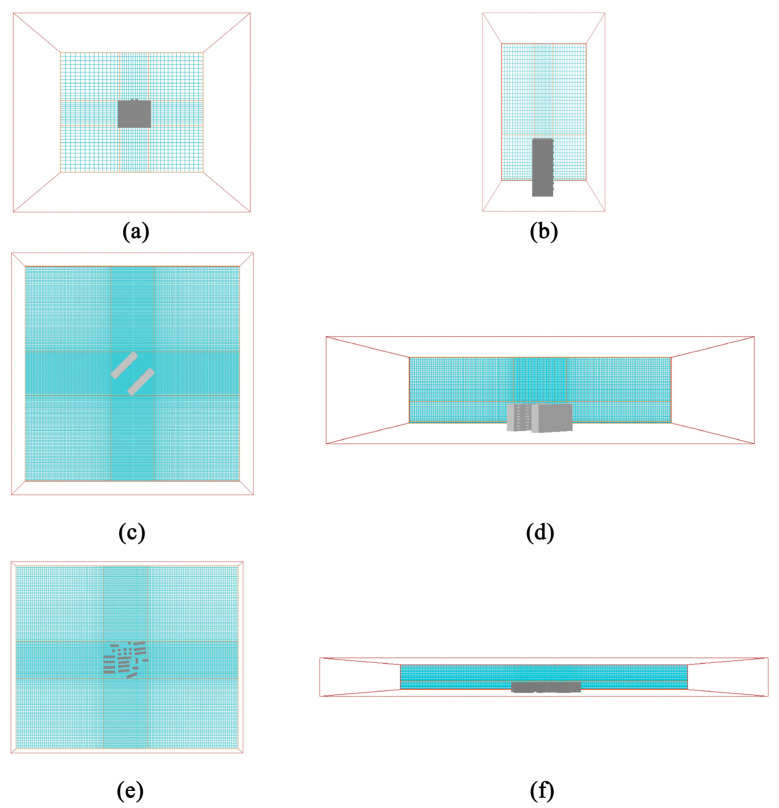
Grid settings: (**a**) top view of single grid; (**b**) side view of single grid; (**c**) top view of townhouse grid; (**d**) side view of townhouse grid; (**e**) top view of community grid; and (**f**) side view of community grid.

Computational domain and grid settings: This study uses the computational fluid dynamics (CFD) method to simulate the wind environment. Its computational domain and grid settings strictly follow relevant specifications and fully consider the balance between calculation accuracy and efficiency. Referring to the “Green Building Evaluation Standard” (GB/T 50378-2019) [59], the boundary of the computational domain extends to 5 times the size of the building model in the length and width directions, and 3 times the height of the model in the height direction. The simulation object is located in the center of the computational domain. Accordingly, the computational domain sizes at the single, townhouse, and community scales are set to 60 m × 50 m × 81 m, 325 m × 325 m × 81 m, and 950 m × 780 m × 81 m, respectively, as shown in Figure 6. In terms of meshing, a sub-region encryption strategy was adopted: in the X and Y-axis planes, the central research object area adopted a 3 m × 3 m fine mesh, the edge area adopted a 5 m × 5 m coarse mesh [69], and the transition zone mesh stretching ratio was 1.2; in the Z-axis direction, a three-section division was carried out, focusing on the pedestrian height wind environment. A 0.5 m × 0.5 m mesh was adopted in the 0–1.5 m height range, a 2 m × 2 m mesh was adopted in the 1.5–27 m building area, and a 3 m mesh was adopted from 27 m to the top of the computational domain. Each section of the mesh was encrypted to the near ground at a stretching ratio of 1.2, to accurately capture the wind field characteristics at the scale of the behavior of the residential street. In order to confirm that the above mesh settings, especially the fine mesh density in the core area, are sufficient to obtain an independent solution that is not affected by the number of meshes, this study then carried out a systematic verification of mesh independence.

**Figure 7:**
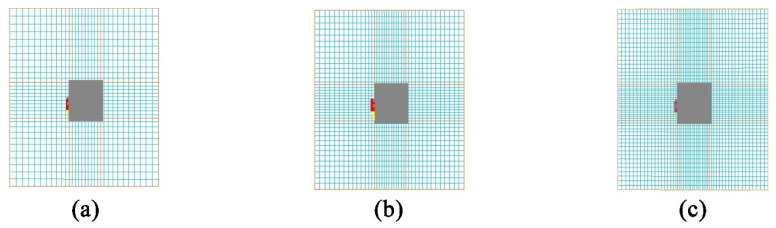
Grid independence verification for (**a**) coarse grid; (**b**) medium grid; and (**c**) fine grid.

Grid independence verification: In order to eliminate the potential impact of grid density on numerical simulation results, this study carried out a systematic grid independence verification. Based on the computational domain of the single building scale, three sets of grid systems were constructed, from coarse to dense. The coarse grid, medium grid, and fine grid were obtained by systematically scaling the basic grid size and adjusting the local encryption strategy. The coarse grid increases the size of the basic grid in all directions to about 1.3 times that of the medium grid and reduces the local encryption level; the fine grid reduces the size of the basic grid to about 0.75 times that of the medium grid and increases the local encryption level. This strategy ensures that the number of grids changes significantly in gradient, as shown in Figure 7, so that the dependence of the numerical solution on the grid can be effectively tested [70]. By focusing on monitoring the characteristic wind speed at the pedestrian height (1.5 m) of the external machine enclosure structure on the windward side of the building, and taking the calculation results of the fine grid as the reference benchmark for analysis, it can be seen that the calculation results of the coarse grid have significant deviations (23.2%), indicating that its grid density is seriously insufficient; meanwhile, the difference between the results of the medium grid and the fine grid is only 2.0%, which is lower than the commonly used allowable threshold in engineering, as shown in Table 4. This proves that the selected medium grid no longer significantly changes the flow field structure around the simulated building, and it has good calculation efficiency, while ensuring calculation accuracy. The result is reliable and economical and is suitable as the final calculation grid for all subsequent simulations, reaching the grid-independent state. 

Reference model verification: Due to the certain differences between the building model and the CAARC prototype dimensions, the reference model is scaled to a depth-to-width ratio range (1.2–1.5) similar to that of the building, to ensure the comparability of key physical phenomena such as flow separation and vortex development. Under the premise of strictly following the original wind tunnel experiment inlet boundary conditions, that is, using the exponential law wind speed profile, the wind speed at the reference height is 12.0 m/s, the roughness index is 0.22, the turbulence intensity is 15%, and the turbulence integral scale is 45 m. The simulation results and experimental data show high consistency: the root mean square error of the average wind pressure coefficient of the building surface is 0.042, and the relative error of 92% of the 25 measuring points is controlled within 5%. Among them, the relative errors of the wind pressure coefficients in key areas such as the center of the windward side, the side edge separation zone, and the center of the leeward side are −3.5%, +3.8%, and −2.2%, respectively. At the same time, the simulation accurately reproduced the flow separation point at 85% of the building height, and the recirculation vortex center position in the wake region at 1.2 H downstream, which is in good agreement with the 1.1–1.3 H range reported in the literature. 

Other working condition settings: Based on the Design Code for Heating, Ventilation and Air Conditioning of Civil Buildings (GB 50736-2012, China) [60] and the local wind conditions of Nanchang City, Jiangxi Province (Longitude 115°27′0″–116°35′0″ E, Latitude 28°09′0″–29°11′0″ N), a summer average wind speed of 3.1 m/s from the west-southwest (WSW) was selected as the inlet boundary condition. The maximum temperature was set to 38 °C under a standard atmospheric pressure of 101,325 Pa. The outlet boundary was defined as a pressure outlet with the pressure gauge set to zero. The simulation was configured to run for 2000 iterations. Furthermore, given that the old urban area of Nanchang is primarily characterized by residential buildings, a ground roughness index of 0.28 was applied. 

## 3. Result

In the study of the ventilation efficiency of the bionic form of the air conditioning outdoor unit enclosure, the effects of five bionic enclosures on the outdoor wind environment were verified from three building scales—single building, townhouse, and building cluster—and compared with the original unstructured outdoor unit. The experimental results mainly include the following three parts.

The first part is a CFD comparative study of five types of bionic envelopes and the original non-envelope at the single building scale.

The second part is a CFD comparative study of five types of bionic envelope structures and the original non-envelope structures at the scale of the townhouse, and at the same time, the experimental data of the building unit scale is combined to extract the bionic envelope structure with the optimal ventilation benefit.

The third part is a CFD comparative study of the original non-envelope structure and the optimal bionic envelope structure at the scale of an old community.

### 3.1. Comparative Study of Bionic Stent Building Units

**Figure 8:**
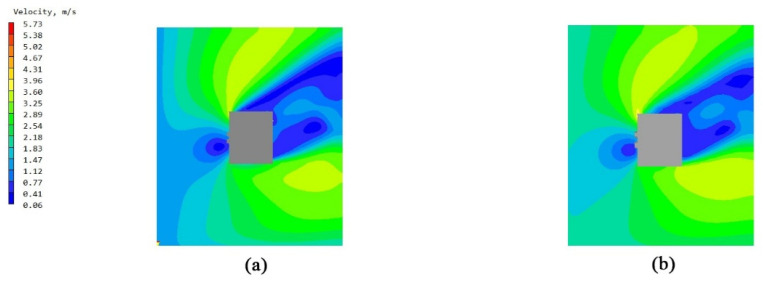
Wind speed diagram of the pedestrian walkway (1.5 m) of the single building with no envelope structure and spider web bionic structure: (**a**) original single wind speed diagram without envelope structure and (**b**) wind speed diagram of a single spider web bionic structure.

**Figure 9:**
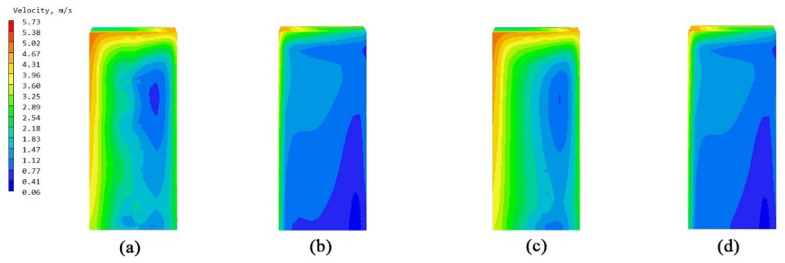
Windward and leeward velocity diagrams: (**a**) windward side velocity diagram of a single unit without an enclosure structure; (**b**) leeward side velocity diagram of a single unit without an enclosure structure; (**c**) windward side velocity diagram of a single unit with a spider web structure; and (**d**) leeward side velocity diagram of a single unit with a spider web structure.

**Figure 10:**
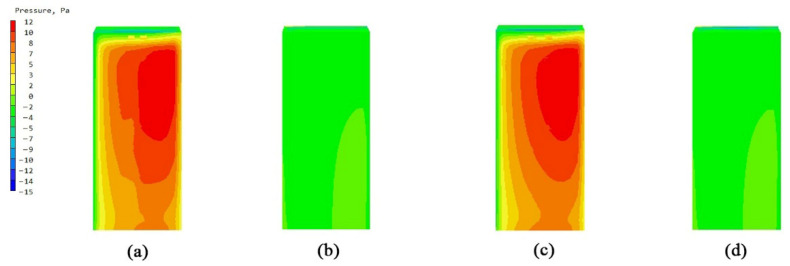
Windward and leeward pressure diagrams: (**a**) wind pressure diagram of the windward side of the unit without an enclosure structure; (**b**) wind pressure diagram of the leeward side of the unit without an enclosure structure; (**c**) wind pressure diagram of the windward side of the unit with a spider web structure; and (**d**) wind pressure diagram of the leeward side of the unit with a spider web structure.

The overall flow field of the building unit was a relatively simple surrounding flow. The models of the non-envelope structure and the five types of bionic envelope structures were imported into the PHOENCIS software for wind environment simulation. After the simulation experiment, it was found that the spider web structure had a more significant impact on the ventilation efficiency of the single building. The simulated wind speed at the pedestrian walkway (1.5 m) of the original structure is shown in Figure 8a,b; the windward and leeward surface speeds of the non-envelope structure are shown in Figure 9a,b, and the wind pressure is shown in Figure 10a,b. The windward and leeward surface speeds of the spider web envelope structure are shown in Figure 9c,d, and the wind pressure is shown in Figure 10c,d. The average wind speed of the six structures at the standard floor height is shown in Table 5.

### 3.2. Simulation Study of Bionic Bracket Structure in Townhouse Buildings

**Figure 11:**
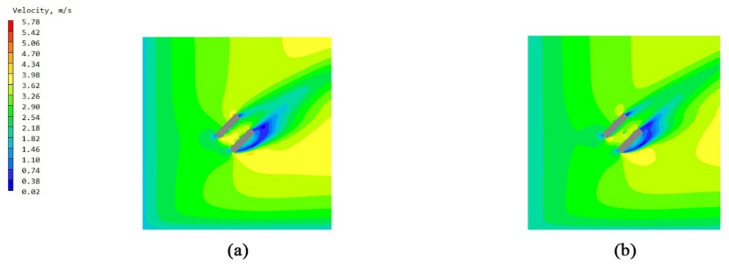
Wind speed diagram of the pedestrian walkway (1.5 m) of the terraced buildings with no envelope structure and spider web bionic structure: (**a**) wind speed diagram of the original terraced buildings with no envelope structure and (**b**) wind speed diagram of the terraced buildings with spider web bionic structure.

**Figure 12:**
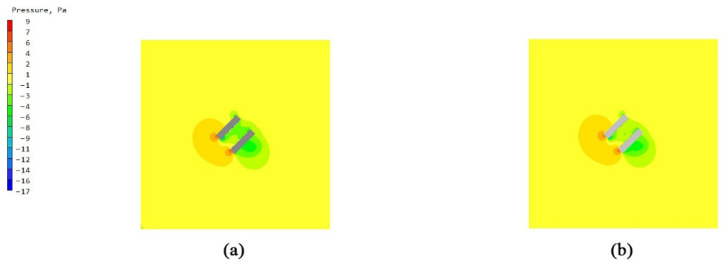
Wind pressure diagram of the pedestrian walkway (1.5 m) of the terraced buildings with no envelope structure and spider web bionic structure: (**a**) wind pressure diagram of the original terraced buildings with no envelope structure and (**b**) wind pressure diagram of the terraced buildings with spider web bionic structure.

The overall flow field of the townhouse was channel flow, and the formation of the corridor caused the narrow tube effect. In order to explore the changes in the efficiency of the air conditioning outdoor unit bionic structure in such a wind environment, two townhouses of the townhouses composed of building units were imported into PHOENCIS software for simulation. After the simulation test, the spider web structure still had the most significant impact on the ventilation efficiency of the townhouse. The simulated wind speed at the pedestrian walkway (1.5 m) of the original structure is shown in Figure 11a,b; the wind pressure is shown in Figure 12a,b; and the average wind speed of the six structures at some standard floor heights is shown in Table 6.

### 3.3. Simulation Study of the Bionic Bracket Structure in an Old Community

**Figure 13:**
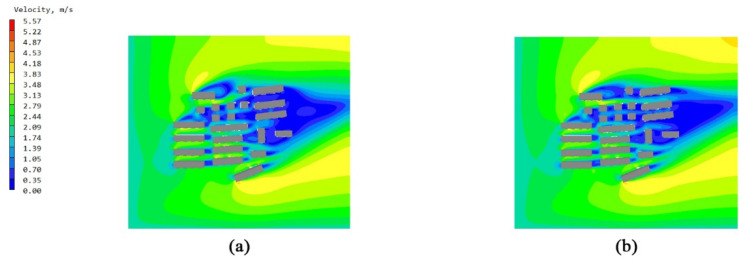
Wind speed diagram of pedestrian walkways (1.5 m) in old communities without envelope structure and with spider web bionic structure: (**a**) wind speed diagram of old communities without envelope structure and (**b**) wind speed diagram of old communities with spider web bionic structure.

**Figure 14:**
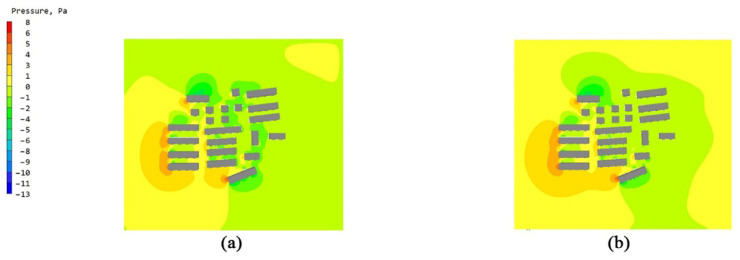
Wind pressure diagram of the pedestrian walkway (1.5 m) of the townhouse houses without envelope structure and with spider web bionic structure: (**a**) wind pressure diagram of the original townhouse houses without envelope structure and (**b**) wind pressure diagram of the townhouse houses with spider web bionic structure.

Due to the characteristics of old communities, such as uneven building orientation, uneven spacing, and cluttered layout, the airflow path became extremely complex, and there were unpredictable separation, rotation, and eddy currents. In order to improve the wind environment, more refined analysis and design were required. The old community models of the non-enclosed structure and the spider web simulation structure were imported into the PHOENCIS software for simulation. The experimental results showed that the spider web structure can still effectively improve ventilation efficiency in the face of the complex wind environment of the community. The wind speed diagrams of the non-enclosed structure and the spider web structure at the pedestrian walkway (1.5 m) are shown in Figure 13a,b, the wind pressure diagrams are shown in Figure 14a,b, and the standard layer wind speed is shown in Table 7.

### 3.4. Investigation of Velocity Vector Fields for Bionic Enclosure Structures

**Figure 15:**
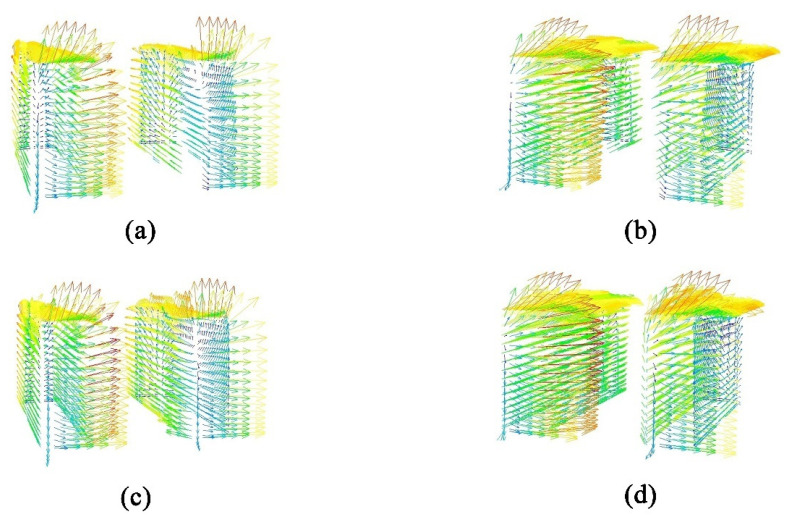
Velocity vector diagrams of wind speed distribution for townhouse buildings with unenclosed configuration and web-inspired bionic structure: (**a**) west windward facade of townhouse buildings with unenclosed configuration; (**b**) south windward facade of townhouse buildings with unenclosed configuration; (**c**) west windward facade of townhouse buildings with web-inspired bionic structure; and (**d**) south windward facade of townhouse buildings with web-inspired bionic structure.

The analysis was conducted based on velocity vector plots obtained from CFD simulations for four configurations. The townhouse-building configuration presented greater complexity in the wind environment, compared to the single-building scale, while maintaining lower computational demands and higher visualization clarity than the full community scale. Therefore, a comparative study of the velocity vector plots was performed for the townhouse-building case. The vector visualization results clearly demonstrate significant differences in airflow organization patterns between the two cases, as shown in Figure 15.

## 4. Discussion

By simulating the summer wind environment of the old community on three scales, the system compared the impact of the original unenclosed structure of the air conditioning outdoor unit and the five types of bionic enclosure structures on the surrounding wind environment. From the simulation’s experimental results of the building unit scale, it can be concluded that the spider web bionic structure is superior to the other four types of structures, in terms of ventilation efficiency and wind speed improvement. Combining Figure 7 and Figure 8, it can be seen that there are a significant dark blue low-speed retention zone and an asymmetric high-speed jet zone on the windward side of the unenclosed structure, and a wide wake low-speed zone is formed at the rear. The corresponding pressure contour in diagram 9 produces a strong positive pressure core on the windward side, while the leeward side shows an obvious pressure gradient and long wake backflow. After the introduction of the spider web enclosure, the deep blue stagnation zone on the windward side is significantly reduced, and the speed of the windward-back side changes from violent discontinuity to a smooth transition; the pressure field shows that the shape of the windward high-pressure zone tends to be scattered. The quantitative results are in good agreement with the above graphs. At six typical heights of 1.5–27 m, the spider web structure slightly increases the average wind speed of each layer compared to the un-enclosed structure. The spider web “cuts” the large-scale inflow into several small-scale jets through the porous grille, thereby shortening the wake, weakening the backflow, and forming a more uniform momentum distribution on the windward side, resulting in a slight increase in the average wind speed of the surface. At the same time, the local pressure drop and multi-point outflow, generated by the enclosure on the windward side, make the overall pressure field smoother. See Table 8.

In the simulation experiment of the townhouse working condition, the airflow channel and the corridor effect play a dominant role in the distribution of the convection field. As shown in Figure 10, a large area of the low-speed retention zone appears on the windward surface and the gap between the building without the enclosure structure. After the airflow enters the corridor, it forms an uneven high-speed jet, and there is a complex vortex structure in some parts. The wake in the leeward area is wider, and the overall organization of the flow field is poor. In Figure 11, the pressure field characteristics show that a concentrated positive pressure peak is formed on the windward side, while there is a wider negative pressure zone on the leeward side, resulting in severe airflow separation. Under the spider web enclosure conditions, the grille plays a “diversion-cutting” role on the incoming flow, and the airflow is decomposed into multiple secondary jets and diffuses in the corridor. The area of the windward stagnation zone is significantly reduced, and the high-speed flow more evenly covers the windward area. The pressure distribution shows that the concentrated high pressure on the windward side is dispersed, the negative pressure range on the leeward side is weakened, and the overall gradient is reduced, which reduces the occurrence of wake suction and local strong recirculation. The numerical results clearly show that the wind speed of the standard floor increases by 0.16–1.76% layer by layer, and the overall average increase is about 1.15%. Compared with the single scale, the improvement is more significant, which proves that the spider web enclosure can effectively improve the airflow distribution and ventilation efficiency in the townhouse and corridor environment. For the air conditioning outdoor unit, this improvement not only helps to maintain the stability of the heat-exchange process, but also may improve the operating life; for the wind environment of the community pedestrian scale, it can reduce the proportion of local stagnant wind areas and improve comfort, as shown in Table 9.

At the overall community scale, the airflow is jointly interfered with by multiple groups of buildings, and the overall flow characteristics are more complex. Combined with Figure 12, it can be seen that under the condition of no enclosure, there is a large range of low-speed retention areas on the windward side, accompanied by a concentrated high-speed jet zone, and the flow field is uneven; on the leeward side, a large low-speed backflow and wake zone are formed. The pressure field in Figure 13 shows that a strong high-pressure core is formed in front of the windward side, while a large area of strong negative pressure is distributed in the leeward area, with a large gradient, inducing obvious separation and eddy currents. After the introduction of the spider web enclosure structure, the area of the windward retention zone is reduced, and the flow field transition is smoother. High-speed flow is no longer concentrated, but it is decomposed by the grille into secondary jets and diffused on a community scale, with a more uniform momentum distribution. The wake range is shortened, and the local vortex is weakened. The pressure distribution characteristics correspond to this; the concentrated high pressure on the windward side is weakened and tends to be scattered, the leeward negative pressure area is reduced, and the overall pressure is closer to the neutral pressure level, indicating that the wake suction is weakened. The comparison results of the wind speed at the standard floor height show that the wind speed of the spider web structure is always higher than that of the non-enclosure condition, with an increase range of 1.55–1.87%, and the overall average increase is about 1.39%. However, the increase at a height of 27 m showed a slight decrease (about −0.4%), indicating that the dominant role of the boundary layer weakened the effect of the spider web enclosure at the upper level. In summary, at the community scale, the spider web structure has a limited effect on the overall wind speed, but it is of great significance in improving the local microenvironment of the middle and lower layers, weakening the wake and pressure gradient, and improving the comfort of the wind environment, as shown in Table 10.

Analysis of the external flow field under the two enclosure configuration schemes for the townhouse buildings reveals distinct characteristics. The vector visualizations of wind speed distribution clearly demonstrate that under the unenclosed condition (Figure 15a,b), the airflow directly impinges on the external unit area, resulting in significant turbulent disturbances. The flow field exhibits chaotic vector orientations and pronounced abrupt changes in velocity/pressure gradients. This not only induces considerable fluctuations in wind load on the building facade, increasing structural stress risks, but also reduces the heat exchange efficiency of the external AC unit due to turbulent interference, thereby accelerating equipment wear. In contrast, for the configuration with the web-inspired enclosure (Figure 15c,d), the pore-guided and constraining effects of the structure promote a more organized airflow pattern. The uniformity of velocity and pressure gradient distributions is markedly improved, achieving effective flow regularization and buffering. This not only mitigates the fluctuation amplitude of building wind loads but also creates a stable heat-dissipation environment for the external AC unit, enhancing operational stability and energy efficiency. In summary, the facade morphology of the web-inspired enclosure demonstrates superior performance in optimizing the local wind environment and balancing structural safety with equipment functionality. 

## 5. Conclusions

Guided by the principles of sustainability, the environmental renewal of old communities in hot–humid regions presents a challenge, necessitating alternatives to large-scale demolition and reconstruction through the micro-renewal of existing buildings. This study focuses on a typical old community in Donghu District, Nanchang. Under the constraint that major updates, such as adjusting building orientation and street width, are difficult to implement, this research adopts a micro-renewal approach via building facade components. Specifically, five types of bio-inspired enclosure structures for air conditioning external units, designed for good ventilation performance, were developed. The wind environment characteristics of the original unenclosed configuration and these bio-inspired enclosures were systematically compared, using computational fluid dynamics (CFD).

The simulation experiments began with the standardized modeling of the old community buildings. Controlled experiments were simulated across three scales: a single building, townhouse buildings, and the entire old community. This process identified the web-inspired structure as the optimal design. The conclusions, discussed in conjunction with aerodynamic mechanisms, are as follows:

1. From the perspective of aerodynamic mechanisms, the porous nature of the web-inspired structure significantly influences the development of the boundary layer on the building facade. Its unique grille design creates a distinct fluid–solid interaction interface, allowing the incoming flow to penetrate the pores while simultaneously facilitating the formation of a stable boundary layer on the building surface. This porous medium effect effectively modulates the flow structure in the near-wall region, delays the onset of flow separation, and improves the pressure distribution characteristics on the building surface. Specifically, it results in a more uniform distribution of the high-pressure zone on the windward side and a significant reduction in the pressure gradient, thereby mitigating the formation of localized strong wind areas.
2. Regarding the vortex structure in the wake region, the web-inspired structure demonstrates a unique capability for turbulence control. Through the flow-rectifying effect of its porous grille, it promotes the breakdown of large-scale, concentrated vortices into multiple smaller-scale, distributed vortex structures. This vortex reorganization mechanism effectively reduces the turbulence intensity in the wake region and improves the air transport characteristics amongst the building group.
3. From the practical standpoint of community micro-renewal, the synergistic effect of multiple web-inspired structures further enhances this outcome, forming a more organized community-scale ventilation network. Quantitative analysis corroborates the above mechanisms: within the typical height range of 1.5–27 m, and benefiting from the optimization of the global flow field organization, the web-inspired structure can increase the average wind speed by approximately 1.1–1.4% at the townhouse building and community scales, with a maximum increase of up to 1.9%. This research confirms the technical feasibility of improving the community wind environment through micro-renewal of attached building enclosures.

In summary, the web-inspired enclosure serves not only as a physical barrier enhancing the structural rigidity for the safety of AC external units, but also as an aerodynamic facility that is capable of actively regulating the local microclimate. By connecting bionic principles with fundamental fluid dynamics, this study reveals the intrinsic mechanism by which porous grille structures optimize the building wind environment. It demonstrates the potential to effectively enhance the quality of the community’s physical environment with minimal intervention, thereby providing a scientific design basis and theoretical support for the sustainable renewal of old communities.

This study also acknowledges several main limitations: the simulation environment neglected the coupling effects of the thermal environment and flow, and did not fully account for the influence of environmental elements such as vegetation. In terms of scope, a systematic parametric study on key parameters of the web-inspired structure (e.g., porosity, pore size) was not conducted. Furthermore, there is a lack of direct comparative validation with field measurement data, including long-term monitoring of wind speed, temperature, noise, and AC energy consumption. Addressing these limitations, future work will prioritize parametric optimization studies of the bio-inspired structures, extend validation efforts to different community morphologies, and employ a combined approach of field monitoring and numerical simulation to establish a comprehensive evaluation system spanning the physical environment to human perception. Quantifying the actual energy-saving benefits will also be a key focus, aiming to provide more reliable technical support for the renewal of old communities. 

## Figures and Tables

**Table 1:** Cost estimation of comprehensive renovation of old residential area renovation.

Comprehensive Renovation of Old Buildings	Main Renovation Content	Unilateral Cost (CNY/m^2^)
Demolition fee		150
Structural reinforcement and transformation	Reinforced for a 30 year service life	1200
Energy-saving transformation	Exterior wall insulation, roof insulation and waterproofing, replacement of rainwater pipes	300
Air conditioning beautification	Air conditioning unit blocking and additional condensate pipe	30
Plastic steel windows and enclosed balconies	Using 60 broken bridge aluminum alloy windows is 280 CNY/m	180
Installation of elevators	Using 60 broken bridge aluminum alloy windows is 280 CNY/m	330
Water supply and drainage pipeline transformation	Main pipe and branch pipe renovation, bathroom and kitchen renovation	400
Strong and weak current transformation	Renovation of public areas and painting of stairwells, railing renewal	400
Heating system renovation	Riser transformation, household metering, radiator replacement	300
Expansion of balcony and bay window	Bay window protruding 600 mm from the exterior wall	700
Total		3990

Note: 1970s 6 story brick–concrete residential building, 4 units, total floor area of approximately 4200 m^2^ calculated.

**Table 2:** Biostructure applications with wind environment optimization benefits.

Biological Structure	Bionic Examples	Structural Function	Studies	Inspiration Extraction
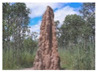	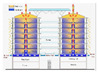	Termite mound ventilation systems.	[39,40,41]	Indoor ventilation of high-rise buildings.
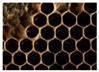	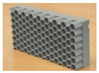	Honeycomb structures for thermal and load management.	[42,43]	The building façade is attached to the vertical greening bionic support.
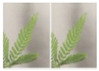	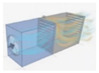	Leaf-inspired surfaces for heat and noise reduction.	[44,45]	Dynamic structure of building facades.
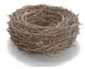	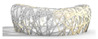	Branch-winding layouts for ventilation and lighting.	[46]	The building frame is strong and lightweight.
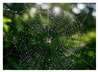	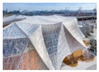	Cobweb structures for performance and ventilation.	[47]	Building façade and roof.
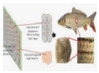	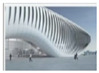	Passive ventilation, solar reflection, and light optimization.	[32,48]	Dynamic structure of building facades.
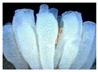	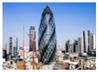	Sponge aerodynamics for wind resistance reduction.	[49]	The overall structure of the building.

**Table 3:** CMAB dataset attribute table.

Attribute Type	Attribute Metrics	Property Description
Geometric properties	Roof shape	Roof profile
	Height	The vertical height of the single building
Indicates the attribute	Function	Residential, office, commercial service, industrial and other functional classifications
	Structure	Frame structure, brick concrete, steel structure, etc.
	Style	Different architectural styles
	Age	Year of construction, 1985–2018 35 categories
	Quality	The maintenance status of the façade, whether it is illegal, etc.

**Table 4:** Coarse, medium, and fine grid characteristics.

Grid Level	Element Count	Characteristic Velocity (m/s)	Relative Change
Baseline Mesh	33,792	1.963	23.20%
Intermediate Mesh	73,964	2.504	2.00%
Refined Mesh	160,113	2.553	Benchmark

**Table 5:** Comparison of wind speed on standard floors of single buildings.

Height (m)	Unenclosed Single	Leaf Townhouse	Honeycomb Single	Cobweb Single	Scales Single	Bird’s Nest Single
1.5	2.225416	2.224171	2.224141	2.238302	2.224175	2.224141
3	2.391976	2.390593	2.390568	2.403999	2.390598	2.390568
9	2.881883	2.880869	2.880854	2.892646	2.880871	2.880871
15	3.205680	3.203293	3.20327	3.213783 3.490931	3.203300	3.203270
21	3.483298	3.480831	3.480784	3.490931 3.994363	3.480831 3.983193	3.480784
27	3.984794	3.983194	3.983130	3.994363	3.983193	3.983130

**Table 6:** Comparison of wind speeds on the standard floors of townhouses.

Height (m)	Unenclosed Townhouse	Leaf Townhouse	Honeycomb Townhouse	Cobweb Townhouse	Scales Townhouse	Bird’s Nest Townhouse
1.5	2.743645	2.761254	2.768233	2.784858	2.767262	2.766421
3	2.804826	2.817279	2.830810	2.846948	2.830820	2.828846
9	3.107364	3.132993	3.143625	3.162149	3.150492	3.140220
15	3.434615	3.295229	3.469199	3.487725	3.472168	3.465668
21	3.764701	3.772811	3.78465	3.796622	3.785625	3.785424
27	4.069164	4.067633	4.070724	4.075769	4.077345	4.071573

**Table 7:** Comparison of wind speed in the standard layer of old communities.

Height (m)	1.5	3	9	15	21	27
Unenclosed community	2.376103	2.434309	2.713332	2.997079	3.302519	3.713194
Cobweb community	2.417698	2.476919	2.763892	3.05129	3.353679	3.698351

**Table 8:** Wind speed gain in the standard layer without envelope structure and spider web structure at the single scale.

Height (m)	Single Scale (Unenclosed)	Single Scale (Cobweb)	Standard Layer Increase
1.5	2.2254	2.2383	+0.58%
3	2.3920	2.4040	+0.50%
9	2.8819	2.8926	+0.37%
15	3.2057	3.2138	+0.25%
21	3.4833	3.4909	+0.22%
27	3.9848	3.9944	+0.24%
Average lift	+0.36%		

**Table 9:** Wind speed gain in the standard layer without envelope structure and spider web structure at the townhouse scale.

Height (m)	Townhouse Scale (Enclosure)	Townhouse Scale (Cobweb)	Standard Layer Increase
1.5	2.7436	2.7849	+1.50%
3	2.8048	2.8469	+1.50%
9	3.1074	3.1621	+1.76%
15	3.4346	3.4877	+1.55%
21	3.7647	3.7966	+0.85%
27	4.0692	4.0758	+0.16%
Average lift	+1.15%		

**Table 10:** Wind velocity gain in standard layer without envelope structure and spider web structure at the community scale.

Height (m)	Community Scale (Enclosure)	Community Scale (Cobweb)	Standard Layer Increase
1.5	2.3761	2.4177	+1.75%
3	2.4343	2.4769	+1.75%
9	2.7133	2.7639	+1.87%
15	2.9971	3.0513	+1.81%
21	3.3025	3.3537	+1.55%
27	3.7132	3.6984	−0.40%
Average lift	+1.39%		

## Data Availability

The original contributions presented in this study are included in the article. Further inquiries can be directed to the corresponding author(s).
